# Distribution Analysis via Mass Spectrometry Imaging of Ephedrine in the Lungs of Rats Orally Administered the Japanese Kampo Medicine Maoto

**DOI:** 10.1038/srep44098

**Published:** 2017-03-08

**Authors:** Takashi Matsumoto, Hirotaka Kushida, Shoko Matsushita, Yoshiyuki Oyama, Takafumi Suda, Junko Watanabe, Yoshio Kase, Mitsutoshi Setou

**Affiliations:** 1 Tsumura Research Laboratories, Kampo Scientific Strategies Division, Tsumura & Co., Ibaraki, Japan; 2 Department of Cellular & Molecular Anatomy, Hamamatsu University School of Medicine, Hamamatsu, Shizuoka, Japan; 3 International Mass Imaging Center, Hamamatsu University School of Medicine, Hamamatsu, Shizuoka, Japan; 4 Second Division, Department of Internal Medicine, Hamamatsu University School of Medicine, Hamamatsu, Shizuoka, Japan

## Abstract

Maoto, a traditional Japanese Kampo medicine, has been used to treat various respiratory diseases, including respiratory infections and influenza. Ephedrine (EPD), the main ingredient in maoto, is also clinically used to treat respiratory diseases. However, the pharmacokinetics and distribution of EPD in the lungs after the administration of maoto have not been demonstrated. This study aimed to determine the concentrations, distribution, and pharmacokinetics of EPD and its precursor methylephedrine (MEPD) in the lungs of rats orally administered maoto (1 and 4 g/kg). We used liquid chromatography–electrospray ionization-tandem mass spectrometry to measure the ingredient concentrations. Both ingredients were detected in maoto-treated lung homogenates. Next, we examined the distribution of both ingredients in lung sections by using matrix-assisted laser desorption/ionization-mass spectrometry imaging, a powerful tool for the visualization of the distribution of biological molecules. The mass spectrometry imaging analysis detected only EPD and provided the first visual demonstration that EPD is distributed in the alveoli, bronchi, and bronchioles in the lungs of rats orally administered maoto (4 g/kg, three times at 2-h intervals). These results suggest that the pharmacological efficacy of maoto for the amelioration of respiratory symptoms is related to the distribution of EPD in the lung.

Maoto is a traditional Japanese Kampo medicine composed of the following four medical plants: the stem of *Ephedra sinica*, the seeds of *Prunus armeniaca*, the cortex of *Cinnamomum cassia*, and the root and stolon of *Glycyrrhiza uralensis.* This medicine has been approved for medical use by the Japanese Ministry of Health and Welfare and has been widely applied to treat various symptoms, such as fever^1^,^2^, malaise^3^, influenza, and upper respiratory infections^4^,^5^,^6^. In addition, the stem of *E. sinica* has traditionally been used to treat asthma^7^.

Ephedrine (EPD), the main ingredient of the stem of *E. sinica*, has been reported to have various pharmacological effects, such as a bronchodilator effect, by stimulating adrenaline β_2_ receptors^8^,^9^; an anti-inflammatory effect, by suppressing the inflammatory cytokine tumor necrosis factor-α (TNF-α)^10^; and a protective effect against inhaled and aspirated materials in the respiratory tract, by increasing ciliary activity^11^. EPD is also produced by metabolizing methylephedrine (MEPD), a precursor of EPD also present in maoto^12^. However, the pharmacokinetics (PK) and distribution of both ingredients in the lungs after the oral administration of maoto have not been previously demonstrated.

In general, the drug concentration in the target tissue is quantified by using various analytical instruments, including liquid chromatography–electrospray ionization-tandem mass spectrometry (LC-ESI-MS/MS), to demonstrate that the administered drug has reached the target organ. In addition, autoradiography analysis is performed to show the distribution of the drug in the target tissue. More recently, matrix-assisted laser desorption/ionization-mass spectrometry imaging (MALDI-MSI) analysis has been developed as a powerful tool for visualization of the distribution of biological molecules or metabolites in tissue sections without radiolabeling^13^,^14^.

The purpose of this study was to determine the concentrations, distribution, and PK of EPD and its precursor, MEPD, in the lungs of rats orally administered maoto. We first used LC-ESI-MS/MS to measure the concentrations of both ingredients in homogenized lungs of rats orally administered maoto, and the PK of these ingredients in the lungs was then evaluated. Next, the distribution of both ingredients in the lung was examined through MALDI-MSI analysis, and subsequent region of interest (ROI) analysis was accomplished based on EPD signal intensity.

## Results

### Identification of EPD and MEPD in the lung

**Figure 1:**
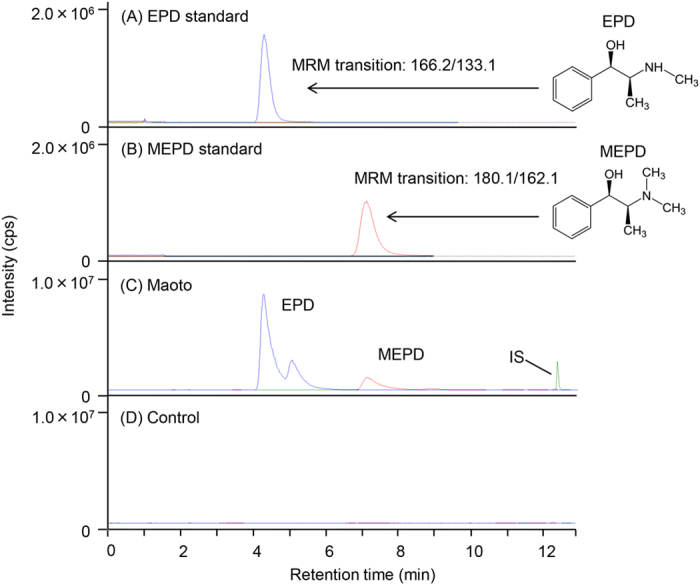
MRM chromatograms of the EPD standard (**A**), MEPD standard (**B**), lung homogenized 0.5 h after oral administration of maoto (**C**), and control lung homogenized before administration of maoto (**D**). IS: vincamine.

Several MRM chromatograms for identification of EPD and MEPD in the lungs of rats orally administered maoto are presented in Fig. 1. Figure 1A shows the chromatogram of an authentic EPD, detected as an MRM transition of *m/z* 166.2/133.1 at a 4.4 min retention time for EPD. Figure 1B shows the chromatogram of authentic MEPD, detected as an MRM transition of *m/z* 180.1/162.1 at a 7.1 min retention time for MEPD. The chromatogram from the lung homogenate obtained 0.5 h after the oral administration of maoto showed peaks with the same MRM transitions and retention times as those in the standard EPD and MEPD chromatograms (Fig. 1C). The peak detected at the rear of EPD was inferred to be the EPD isomer pseudoephedrine, which has been found to be present in maoto in preliminary experiments. However, these peaks were not observed in the non-treated lung homogenate control (Fig. 1D). From these results, the peaks observed in the homogenate of lung after the maoto administration were identified as EPD and MEPD. As shown in Fig. 1C, the peak with an MRM transition of *m/z* 355.3/337.1 for the vincamine used as an internal standard (IS) was detected at 12.4 min retention time, and this peak was not affected by administration of maoto and did not interfere with detection of EPD or MEPD.

### PK of EPD and MEPD in the lungs

**Figure 2:**
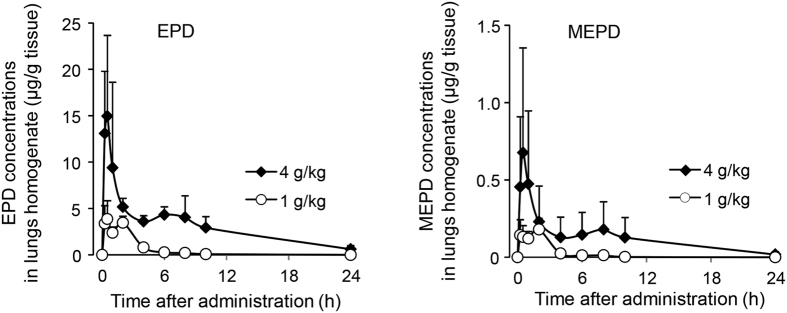
The lung concentration–time curves of EPD and MEPD after oral administration of maoto. Rats (n = 3 animals/each time point) were sacrificed at 0 (pre-administration), 0.25, 0.5, 1, 2, 4, 6, 8, 10, and 24 h after oral administration of maoto (1 and 4 g/kg). Lung homogenate concentrations of EPD and MEPD were measured by LC-ESI-MS/MS analysis. The quantitation limits of EPD and MEPD were 5 and 0.1 ng/g tissue, respectively. Each value represents the mean ± S.D.

The time-dependent changes in EPD and MEPD concentrations in the lungs of rats orally administered maoto (1 and 4 g/kg) are presented in Fig. 2, and the PK parameters calculated from those data are presented in Table 1. Both ingredients exhibited a bimodal distribution. The first peaks appeared at 0.25 to 0.5 h, and the second peaks were prolonged from 2 h to 6 or 8 h after the dose of maoto was increased. The maximum concentration (*C*_max_) values of both ingredients were increased in a dose-dependent manner, and the EPD values were increased by approximately 20–fold compared with MEPD. The apparent elimination half-life (*t*_1/2_) values of both ingredients were prolonged by increasing the dose of maoto, i.e., 1 to 2 h at the low dose and 4 to 6 h at the high dose. The area under the lung concentration–time curve from zero to last observation time (AUC_*0–last*_) values of both ingredients increased in a dose-dependent manner.

### Distribution of EPD in the lung

**Figure 3:**
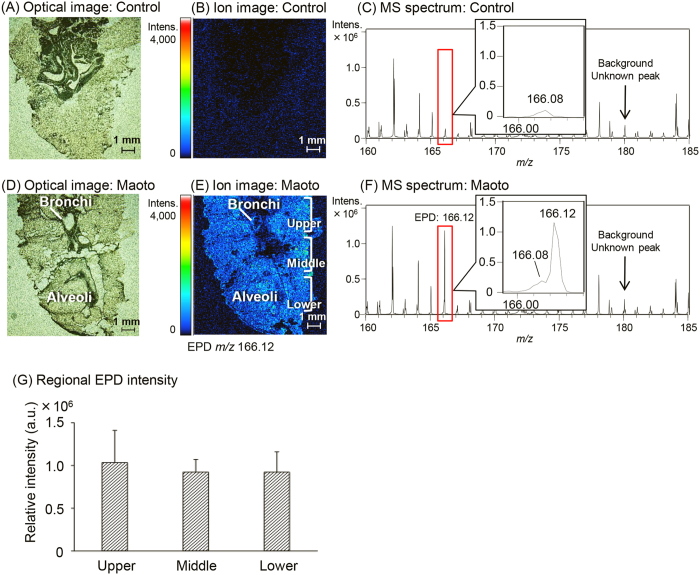
Ion images and MS spectra in the lung sections of non-treated control and maoto-treated rats. (**A**), (**B**), and (**C**) present the optimal image, ion image, and MS spectrum in the lung sections of the control rats, respectively, and (**D**), (**E**), and (**F**) represent those of the maoto-treated rats. The ion images were acquired with 50-μm spatial resolution and show the peak for the protonated signal (*m/z* 166.12: [M + H]^+^) of EPD. The intensities in the ion images ranged from 0 to 4,000 a.u. In three regions divided into the upper, middle, and lower parts (**E**), six areas (0.5-mm square/each area) in each region were selected by using Imaging MS Solution software, and then the average signal intensity of *m/z* 166.12 in each square was calculated (**G**). Scale bar: 1 mm. Each data point represents the mean ± S.D. One-way ANOVA revealed no significant differences among the three regions [*F*(2, 15) = 0.34, *p* = 0.715].

MALDI-MSI analysis was performed by using lung samples obtained 1 h after oral administration of maoto to clarify the EPD and MEPD distribution in the lungs of rats treated with maoto (Fig. 3). Bronchi and alveoli were observed in the optical images of the lung sections (Fig. 3A and D). When MALDI-MSI analyses were examined by detection of the protonated molecules ([M + H]^+^: *m/z* 166.12 and 180.13, respectively) of EPD (MW: 165.24) and MEPD (MW: 179.26), the EPD-derived signals were observed throughout the maoto-treated lung sections, including in the alveoli and bronchi (Fig. 3E). However, none of the signals were observed in the control tissue sections (Fig. 3B). To clarify the EPD distribution in the lungs of rats orally administered maoto, the lung sections were divided into three regions (upper, middle, and lower parts as shown in Fig. 3E). Then, the regional EPD signal intensity was compared through ROI analysis (Fig. 3G). No significant differences were observed in the signal intensities of protonated EPD (*m/z* 166.12: [M + H]^+^) among the three regions. However, the MEPD-derived signals were observed in neither the control nor the maoto-treated lung sections (data not shown here). MS spectrum scanning from *m/z* 100 to 200 showed that the strong protonated signal (*m/z* 166.12) of EPD was present only in the maoto-treated tissue section (Fig. 3F). The weak intensity resembling the protonated signal (*m/z* 180.13) of MEPD was observed in the non-treated control tissue section (Fig. 3C), but this spectrum was not enhanced, even in the maoto-treated tissue section (Fig. 3F).

Next, to verify that the visualized signal (*m/z* 166.12) detected in the maoto-treated lung section was EPD, the MS/MS spectrum of the signal was obtained by using the same tissue section. The spectrum of the authentic EPD (Supplementary Figure S1) revealed the EPD-derived specific fragment ion (*m/z* 148.1) dehydroxy-EPD, but not the precursor ion of EPD. The spectrum of maoto-treated lung sections (Supplementary Figure S1) also revealed the same fragment ion with the authentic EPD, thus indicating that the visualized signal detected in the maoto-treated lung sections was EPD.

**Figure 4:**
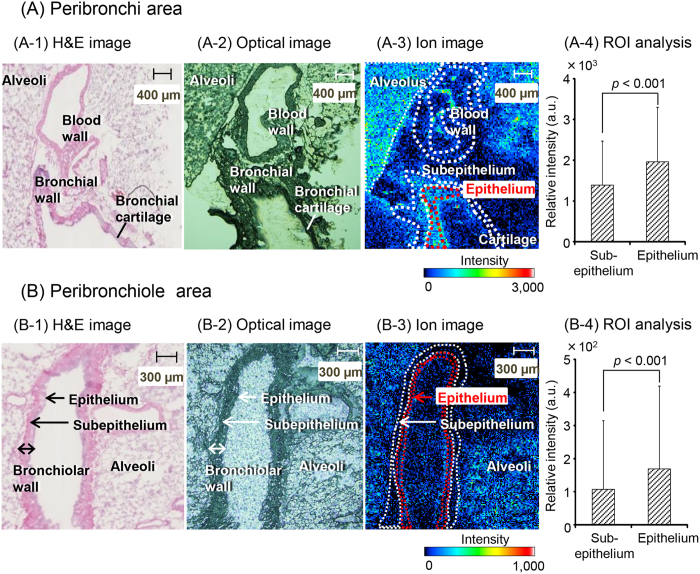
EPD ion images in the peribronchial and peribronchiole area . The cell morphologies of the peribronchi area are shown as H&E (**A-1**), optimal (**A-2**) and ion images (**A-3**) (30 μm spatial resolution). Furthermore, the signal intensities of protonated EPD (*m/z* 166.12) in the epithelium area (in red dotted line) and subepithelium area, including smooth muscle (in white dotted line), were evaluated by using Imaging MS Solution software in the ROI analysis (**A-4**). Similar images and an ROI analysis in the peribronchiole area are presented in (**B-1**), (**B-2**), (**B-3**) (15 μm spatial resolution), and (**B-4**). The intensities in the ion images ranged from 0 to 3,000 (**A-3**) and 0 to 1,000 (**B-3**) a.u. Each value in (**A-4**) and (**B-4**) represents the mean ± S.D. *p* < 0.001 (Student’s *t*-test). Scale bar: 400 μm (peribronchial area) and 300 μm (peribronchiole area).

Next, the detailed distribution or localization of EPD in the peribronchial and peribronchiole areas were further examined (Fig. 4). The cell morphologies of the alveoli, blood vessel wall, bronchial cartilage, bronchial wall, and bronchiolar wall in the optimal image (Fig. 4A-2 and B-2) were confirmed by comparison with those in the image of hematoxylin and eosin (H&E) stained tissue (Fig. 4A-1 and B-1). The ion images of the peribronchial and peribronchiole areas are presented in Fig. 4A-3 and B-3, respectively. These images indicated that strong protonated EPD signals (*m/z* 166.12) were present in the alveoli, bronchial wall, and bronchiolar wall but not in the blood vessel wall and bronchial cartilage. Furthermore, the intensities in the epithelium area (in red dotted line) and subepithelium area, including the smooth muscle (white dotted line) of bronchial or bronchiolar wall, were measured (Fig. 4A-3 and B-3). The ROI analysis of the bronchial or bronchiolar wall indicated that the signal intensity in the epithelium area was significantly higher than that in the subepithelium area, including the smooth muscle (Fig. 4A-4 and B-4).

## Discussion

In this study, MRM analysis using LC-ESI-MS/MS of EPD and MEPD in the lung homogenates demonstrated that both ingredients reached the lung after the oral administration of maoto. In the PK experiments, the *t*_max_ values of EPD and MEPD were both relatively fast; both ingredients reached the lung within 0.25 to 0.5 h after the oral administration of maoto. Ono *et al*. have also demonstrated that orally administered EPD is promptly absorbed into the plasma in rats^15^. Together, these findings indicate that both ingredients in maoto are quickly absorbed into the blood and then reach the lung after the oral administration of maoto. The lung concentration–time curves for both ingredients after the oral administration of maoto exhibited a bimodal distribution. However, such a bimodal pattern has not been observed in the plasma when EPD is orally administered to rats^15^. Although the reason was unclear in this study, the bimodal distribution has been reported to be caused by several factors, such as the coexistence of the precursor^16^, enterohepatic circulation^17^, and plural absorption regions (small and large intestines)^18^ for the ingredients in the Kampo medicine. Why the second peaks of the lung concentrations of both ingredients were delayed with an increasing the dosage of maoto remains unclear. Further experiments are needed to verify the bimodal distribution.

The *C*_max_ and AUC_*0-last*_ value of EPD increased by approximately 20–fold compared with MEPD for any of the doses. Differences in the amounts of the ingredients in the maoto extract (EPD: 10.4 mg/g, MEPD: 0.804 mg/g) may explain this result. In addition, the finding that the concentration ratio of both ingredients (*C*_max_ ratio of EPD/MEPD = 22) in the lung was greater than that (content ratio, EPD/MEPD = 13) in the extract suggests that MEPD metabolism may have been involved, because MEPD is metabolized to EPD *in vivo*^12^. Furthermore, the *C*_max_ and AUC_*0-last*_ values of both ingredients increased in a dose-dependent manner. These results indicated that EPD and MEPD were absorbed in the blood and lungs without saturating within the dose range of this study.

MALDI-MSI analysis used to clarify the distribution or localization of EPD and MEPD in the lungs is a unique procedure in which the localization of biological molecules or metabolites can be visualized in the tissue section^19^,^20^,^21^. In the present study, we provided the first visual verification that the studied maoto ingredients reached the lung, by detecting and visualizing EPD signals in the lung sections of rats orally administered maoto (4 g/kg, three times). However, we were unable to detect the specific molecular signals for MEPD in the lung sections. Because MEPD was detected in the lung homogenate sample (Fig. 1), our inability to detect MEPD in the MALDI-MSI analysis may have been that the limit of detection was influenced by the direct ionization of molecules in the tissue sections and contaminants, such as proteins and lipids, inhibited ionization of the target ingredient and led to reduced sensitivity^22^. A more sensitive analytical method is needed in the future to accurately determine the distribution in the tissue of low-density ingredients, such as MEPD.

Therefore, in this study, we focused on EPD and performed more detailed MALDI-MSI analysis. The MS/MS spectrum of the maoto-treated tissue section demonstrated that the EPD specific product ion (*m/z* 148.1) was generated from the precursor ion (*m/z* 166.12) and that of the authentic EPD, thus indicating that the signals in the ion image were related to EPD.

The ROI analysis showed no significant differences among the three regions, thus suggesting that EPD is almost uniformly distributed in the lungs of rats receiving maoto.

EPD after the oral administration of maoto was demonstrated for the first time to be distributed in the alveoli (Fig. 3), which occupies 85% of the lung volume^23^. Because EPD has anti-inflammatory effects by suppressing the inflammatory cytokine TNF-α^10^, the distribution of EPD in the alveoli is likely to be involved in these anti-inflammatory effects.

Furthermore, ion image analysis in the peribronchi area indicated that EPD was distributed in both the epithelial and subepithelial areas, including the smooth muscle of the bronchi (Fig. 4A). Adrenergic compounds, such as EPD, increase in ciliary activity^11^. The cilia play an important role in the protective response, which is similar to the sputum action response, to inhaled foreign substances^24^. Therefore, the distribution of EPD in the bronchial epithelium suggests that maoto might defend against respiratory tract infections by increasing ciliary activity. In addition, adrenaline β_2_ receptors involved in bronchodilator action are present in the bronchial smooth muscle^25^. Therefore, MALDI-MSI analysis demonstrating that EPD was distributed in the subepithelium area, including the smooth muscle of the bronchi (Fig. 4A) may support the previously described mechanism in which EPD dilates the bronchi via stimulation of the β_2_ receptors^7^,^8^.

In this study, EPD was also demonstrated to be localized to the bronchiolar epithelium (Fig. 4B). Clara cells, a nonciliated type of cell found in the epithelial monolayer of terminal bronchioles, secrete pulmonary surfactants that have an important role in maintaining the structure of the lung^26^. The β_2_ receptor agonist facilitates secretion of surfactant, thus leading to apoptosis of eosinophils involved in the induction of asthma or related inflammation^27^. These findings together with our results demonstrating the localization in bronchiolar epithelium of EPD suggest that the anti-inflammatory effect of EPD is caused by increased secretion of pulmonary surfactant in addition to suppression of TNF-α, as described above.

EPD is present in several Kampo medicines prescribed to treat respiratory disease, such as Makyokansekito and Gokoto^28^. Our approaches could potentially be used to determine the tissue distribution of ingredients in several kampo medicines.

In this study, PK experiments using LC-ESI-MS/MS analysis demonstrated that the active ingredients, EPD and MEPD, reached the lung, which is the pharmacological target organ of maoto, and EPD concentrations in lung homogenates were higher than those of MEPD after the maoto administration. Furthermore, the distribution or localization of EPD was demonstrated visually in tissue sections of the lung through MALDI-MSI analysis. These results support the pharmacological action of this medicine and may be useful in future pharmacological and PK studies on various Kampo medicines.

## Methods

### Drugs and reagents

The dry powdered extract of maoto (Lot No. 331010700) used in the present study was supplied by Tsumura & Co. (Tokyo, Japan). The extract was produced by a unique method without medicinal additives. The maoto was composed of the following four dried crude components in the percentages indicated in parentheses: the stem of *E. sinica* (32.3%), seeds of *P. armeniaca* (32.3%), the cortex of *C. cassia* (25.8%), and the root and stolon of *G. uralensis* (9.7%). The mixture of four crude components was extracted into purified water at 95 °C for 1 h, and the extraction solution was separated from the insoluble waste and concentrated by removing solvent under reduced pressure. Spray drying was used to process the dried extracted powder. We previously confirmed in-house that 1 g of maoto extracted powder contained 10.4 mg EPD and 0.804 mg MEPD by an LC-ESI-MS/MS analysis.

Authentic *l*-EPD and *dl*-MEPD were purchased from Alps Pharmaceutical Ind. Co. (Gifu, Japan). α-Cyano-4-hydroxycinnamic acid (CHCA) and vincamine were purchased from Bruker Daltonics (Billerica, MA, USA) and Tokyo Chemical Ind. Co. (Tokyo, Japan), respectively. Other chemicals were purchased from commercial sources.

### Animals

Male Sprague–Dawley rats were purchased from Japan SLC (Shizuoka, Japan). The animals were housed at a temperature of 23 ± 3 °C, relative humidity of 50 ± 20%, and a 12-h light/dark cycle with lights on from 07:00–19:00 h daily. Rats were allowed free access to water and standard laboratory food (MF, Oriental Yeast Co., Ltd., Tokyo, Japan). After habituation for 1 week, 7- to 8-week-old rats were used in this study.

This study was approved by and conducted according to the “Guidelines for the Care and Use of Laboratory Animals” of the Laboratory Animal Committee of Tsumura & Co. (permit no: 14-007 and 15–058). All surgery was performed under isoflurane anesthesia, and all efforts were made to minimize suffering.

### PK analysis

Maoto (1 or 4 g/kg) prepared by dissolution in 10 mL of distilled water was orally administered to 16-h fasted rats. These animals (n = 3/each point) were sacrificed at 0 (pre-administration), 0.25, 0.5, 1, 2, 4, 6, 8, 10, and 24 h after the oral administration of maoto by exsanguination from the abdominal inferior vena cava, and were subsequently perfused with saline. The lungs were immediately removed, frozen on dry ice powder, and stored at –80 °C until used for analysis.

On the day of analysis, the frozen lung was thawed at room temperature and homogenized by using a homogenizer (IKA-T10 model; IKA, Staufen, Germany) after the addition of four volumes (v/w) of 50% acetonitrile. The homogenate (200 μL) was mixed with 25 μL of methanol, an equal volume of vincamine (10 ng/mL) as an IS for EPD and MEPD, and 250 μL of ethyl acetate. In the preparation of the calibration curve, the same volumes of various concentrations of working solution were used instead of the methanol. The mixture was centrifuged at 7,000 × *g* and 4 °C for 5 min. The supernatant was dried at 40 °C under a stream of nitrogen gas. The dried residue was dissolved in 50 μL of the initial mobile phase used for the LC-ESI-MS/MS system, and then a 10-μL portion was injected into the LC-ESI-MS/MS system for quantification of EPD and MEPD.

The following LC-ESI-MS/MS analytical conditions were used: samples injected into the LC-ESI-MS/MS system were chromatographically separated on an Agilent 1290 Infinity LC system (Agilent Technologies, Santa Clara, CA, USA) with an Inertsil Ph-3 column (100 × 2.1 mm I.D., 3-μm particle size; GL Sciences, Tokyo, Japan) at 40 °C. The mobile phase consisted of solution A (0.2% formic acid, v/v) and solution B (acetonitrile) with a gradient of solution B (2%, 0 min; 2%, 9 min; 90%, 13 min; 2%, 13.01 min; 2%, 18 min; v/v) at a flow rate of 0.3 mL/min. A Triple Quad 6500 mass spectrometer fitted with a TurboIonSpray electrospray ionization interface (AB Sciex, Tokyo, Japan) was used for mass spectrometry. The mass spectrometer was operated in positive-ion mode. The high-purity nitrogen gas was composed of ion source gas 1, ion source gas 2, curtain gas, and collision-activated dissociation gas at pressures of 50, 40, 30, and 8 psi, respectively. The optimized TurboIonSpray voltage and temperature were set at 4500 V and 600 °C, respectively. EPD (MW: 165.24), MEPD (MW: 179.26), and vincamine (MW: 354.45) were analyzed by multiple reaction monitoring (MRM) transitions at *m/z* 166.2 to 133.1, 180.1 to 162.1, and 355.3 to 337.1 (precursor ion: [M + H]^+^ to representative product ion), respectively.

In quantitative analysis of ingredients in Kampo medicine containing various ingredients, the compound that is not included in targeted kampo medicine is often selected as IS. In this study, vincamine, which was previously confirmed to be not included in maoto by LC-MS/MS analysis, was used as IS. The recovery rate after pretreatment with vincamine was 56.8%, and the CV was 1.0%. EPD and MEPD concentrations in the lung were calculated from calibration curves prepared in analyst software (version 1.6.2, AB Sciex). The linearity range of the calibration curve was 5 to 2000 ng/g tissue (correlation coefficient: *r = *0.999) for EPD or 0.1 to 100 ng/g tissue (*r* = 0.998) for MEPD. When the concentration of the ingredient in the sample exceeded the calibration range, it was re-measured by using a diluted sample to be measured within the calibration ranges.

Pharmacokinetic constants, including the *C*_max_, *t*_max_, *t*_1/2_, and AUC_*0–last*_, were analyzed by noncompartmental modeling using Phoenix WinNonlin (version 6.3, Certara L.P., St. Louis, MO, USA). The *t*_1/2_ was divided by log_e_2/*k*e, where *k*e is the terminal elimination (at least three data points on the descending linear limb) rate constant.

### Distribution analysis

Maoto (4 g/kg, n = 4) or distilled water (10 mL/kg, n = 4) as the vehicle control was orally administered three times every 2 h to the 16 h-fasted rats. These rats were sacrificed 1 h after the final administration by exsanguination from the abdominal inferior vena cava, and were subsequently perfused with saline. After the lungs with bronchi were removed, saline from the bronchi was injected to inflate the lungs. The expanded lungs were immediately frozen on dry ice powder and then stored at –80 °C until used for analysis.

On the day of analysis, the frozen tissue was longitudinally cut into 8-μm-thick sagittal sections at −20 °C in a cryo-microtome (Leica CM1950; Leica Microsystems, Wetzlar, Germany). Serial sagittal sections prepared from one of the pulmonary lobes were thaw mounted onto indium–tin-oxide coated slides (Matsunami Glass Ind., Osaka, Japan) pre-coated with 200 μL of matrix solution (20 mg/mL CHCA in acetonitrile:2-propanol:0.1% trifluoroacetic acid = 40:10:50, v/v/v) by using an air-brush (Procon Boy FWA platinum; Mr. Hobby, Tokyo, Japan), and then 800 μL of the matrix solution was sprayed evenly over the tissue sections on the slides for MALDI-MSI analysis. Additionally, some of the serial sections were thaw-mounted onto standard microscope glass slides (Matsunami Glass Ind.) for H&E stain.

MALDI-MSI analysis was performed by using a Mass Microscope, a prototype of iMScope equipped with a 355-nm Nd:YAG laser (Shimadzu, Kyoto, Japan) in positive-ion mode^29^. MSI measurements were performed with 15 to 50 μm spatial resolution. The sample voltage and detector voltage were set at 3.5 kV and 2.1 kV, respectively. Spectra were acquired in the *m/z* 100 to 200 range by using a laser energy of 35 to 40 μJ. The laser irradiated each position 100 times with a 10- to 25-μm diameter and a 1-kHz repetition rate. The ion images of EPD and MEPD corresponded to the protonated molecular ions ([M + H]^+^) at *m/z* 166.12 and 180.13. The collision energy was optimized to obtain an intense pattern of fragment ions for EPD. The distributions of EPD in the upper, middle, and lower regions of the lung section were evaluated by ROI analysis, in which the signal average of six areas (0.5-mm square/each area) selected in each region was evaluated by using Imaging MS Solution (version 1.12, Shimadzu). In the ROI analysis between the epithelium and subepithelium in the bronchial or bronchiolar wall, the EPD signal average in each region surrounded by a frame was evaluated by using Imaging MS Solution software.

### Statistical analysis

The ROI analysis data are expressed as the mean ± standard deviation (S.D.). The statistical analysis among three regional distributions of EPD in the lung was evaluated by performing one-way analysis of variance (ANOVA) using SAS 9.2 software (SAS Institute, Inc., Cary, NC, USA). The intensities of EPD were compared between the epithelium and subepithelium in the bronchial or bronchiolar wall by using Student’s *t*-test. The significance threshold in each statistical analysis was *p* < 0.05.

## Additional Information

**How to cite this article**: Matsumoto, T. *et al*. Distribution Analysis via Mass Spectrometry Imaging of Ephedrine in the Lungs of Rats Orally Administered the Japanese Kampo Medicine Maoto. *Sci. Rep.* **7**, 44098; doi: 10.1038/srep44098 (2017).

**Publisher's note:** Springer Nature remains neutral with regard to jurisdictional claims in published maps and institutional affiliations.

## Supplementary Material

Supplementary Information

## Figures and Tables

**Table 1:** The pharmacokinetic parameters of EPD and MEPD after oral administration of maoto.

Compound	Dose (g/kg)	1st peak (h)	2nd peak (h)	*t*_max_ (h)	*C*_max_ (μg/g)	AUC_*0-last*_(μg·h/g)	*t*_1/2_ (h)
EPD	1	0.5	2	0.5	3.86	11.9	1.56
	4	0.5	6	0.5	14.9	75.2	6.15
MEPD	1	0.25	2	2	0.179	0.542	1.50
	4	0.5	8	0.5	0.676	3.12	4.82
