# Supplementary material for: Distribution Analysis via Mass Spectrometry Imaging of Ephedrine in the Lungs of Rats Orally Administered the Japanese Kampo Medicine Maoto
Source: Sci Rep. 2017 Mar 8;7:44098. doi: 10.1038/srep44098 (PMC5341069; doi:10.1038/srep44098)
Supplement: Supplementary Information [file srep44098-s1.pdf]

Title

Distribution Analysis via Mass Spectrometry Imaging of Ephedrine in the Lungs of Rats Orally Administered the Japanese Kampo Medicine Maoto

Authors

Takashi Matsumoto[1,*], Hirotaka Kushida[1], Shoko Matsushita[2,3], Yoshiyuki Oyama[4], Takafumi Suda[4], Junko Watanabe[1], Yoshio Kase[1], and Mitsutoshi Setou[2,3]

1) Tsumura Research Laboratories, Kampo Scientific Strategies Division, Tsumura & Co., Ibaraki, Japan

2) Department of Cellular & Molecular Anatomy, Hamamatsu University School of Medicine, Hamamatsu, Shizuoka, Japan

3) International Mass Imaging Center, Hamamatsu University School of Medicine, Hamamatsu, Shizuoka, Japan

4) Second Division, Department of Internal Medicine, Hamamatsu University School of Medicine, Hamamatsu, Shizuoka, Japan

* Author to whom correspondence and reprint requests should be sent:

Takashi Matsumoto

Tsumura Research Laboratories, Tsumura & Co.

3586 Yoshiwara, Ami-machi, Inashiki-gun, Ibaraki 300-1192, JAPAN

Phone: +81-29-889-3852, Fax: +81-29-889-3870

E-mail: matsumoto_takashi@mail.tsumura.co.jp

(A) MS/MS spectrum of authentic standard substance EPD

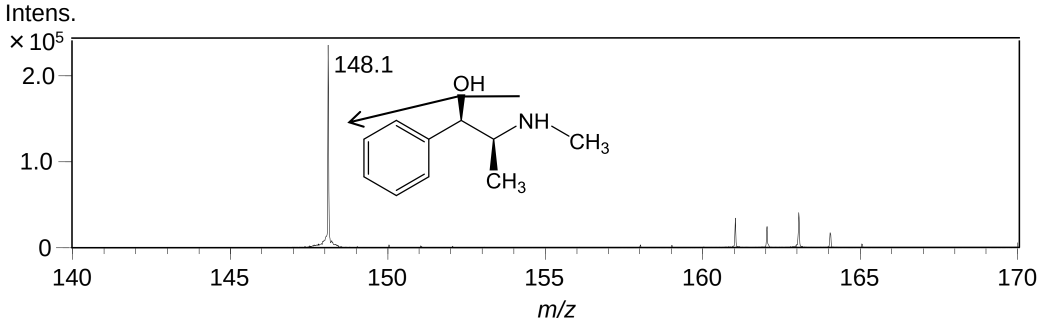

(B) MS/MS spectrum of EPD in the lung treated with maoto

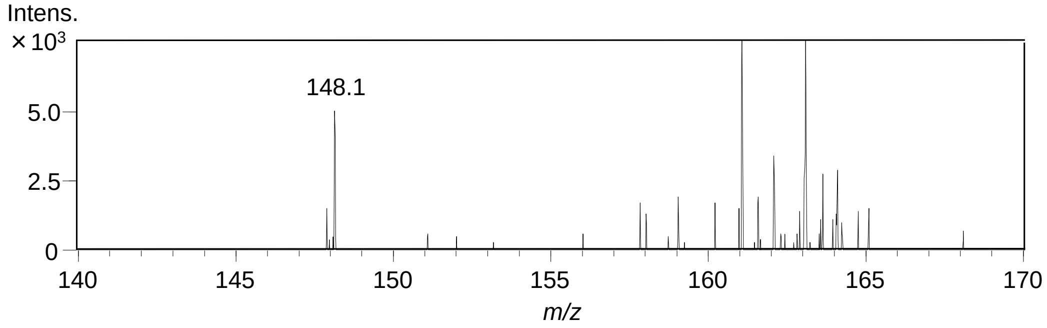

Supplementary Figure S1. MS/MS spectra in an authentic EPD (A), and the lung section of maoto-treated rats (B). The fragment ions were produced by collision-induced dissociation of the EPD precursor ion (*m/z* 166.12). The EPD-derived specific fragment ion (*m/z* 148.1) was present in both spectra.
